# Compact AMC-Backed Flexible UHF RFID Tag Antenna for On-Body Biomedical Applications

**DOI:** 10.3390/s26061922

**Published:** 2026-03-18

**Authors:** Aarti Bansal, Giovanni Andrea Casula

**Affiliations:** 1 Department of Electronics and Communication Engineering, Thapar Institute of Engineering and Technology, Bhadson Road, Patiala 147004, Punjab, India; aarti.bansal@thapar.edu; 2 Department of Electrical and Electronic Engineering (DIEE), University of Cagliari, 09123 Cagliari, Italy

**Keywords:** UHF-RFID system, AMC metasurface, flexible antennas, on-body applications, biomedical applications

## Abstract

**Highlights:**

**What are the main findings?**

- A miniaturized AMC metasurface utilizing meandered Jerusalem-cross and interdigitated comb-like features was developed, achieving a 50% size reduction and an ultra-compact footprint (0.0246 λ^2^) compared to conventional unit cell geometries.
- The integration of the AMC shielding layer results in a substantial gain enhancement of approximately 13 dB and a reduction in SAR values by more than an order of magnitude compared to standalone tags on the human body.

**What are the implications of the main findings?**

- The use of a high-permittivity, silicon-doped biocompatible substrate enables a flexible and low-profile design (2.23 mm) suitable for epidermal sensing of physiological parameters such as pH, temperature, and skin impedance.
- The design’s robust platform tolerance and stability under bending ensure reliable long-range communication and operational integrity across diverse anatomical regions and dynamic wearable environments.

**Abstract:**

This paper presents the design, modeling, and numerical validation of a compact artificial magnetic conductor (AMC)–backed flexible UHF RFID tag antenna intended for on-body biomedical and wearable sensing applications. Human tissue proximity typically causes severe detuning, radiation efficiency degradation, and increased specific absorption rate (SAR) for conventional RFID tag antennas. To address these limitations, a miniaturized AMC metasurface based on a modified Jerusalem-cross geometry with meandered and interdigitated features is developed on a high-permittivity biocompatible substrate using CST Studio Software (2025). Full-wave simulations demonstrate that the proposed design, with an ultra-compact footprint of 0.0246 λ^2^ (32.12 mm × 64.24 mm), functions as an effective shielding element, significantly enhancing the tag antenna gain and reading range by an order of magnitude compared to conventional on-body tags, while simultaneously reducing backward radiation and SAR. The antenna demonstrates robust platform tolerance and excellent isolation from the human body, ensuring high reliability. Fabricated on a thin, flexible, biocompatible, silicon-doped dielectric substrate, this device also functions as an epidermal antenna for on-skin health parameter sampling. This research paves the way for advanced, non-invasive wearable medical devices with superior performance.

## 1. Introduction

The rapid digitization of healthcare has catalyzed a paradigm shift toward personalized medicine, centered on wearable biomedical devices that enable continuous, non-invasive health monitoring and diagnostics [1,2]. These innovations have brought new design challenges for wearable, implantable, and biocompatible systems (acting as the interface between the human body and the digital world) that rely on electromagnetic (EM) wave propagation to gather data about a person’s emotional, behavioral, environmental, and physical states, ultimately enhancing overall well-being [3,4]. As these devices evolve at the intersection of materials science, wireless communication, electromagnetics, mechanics, and electronics, there is an urgent need for radiators that are not only high-performing but also mechanically conformable and electromagnetically robust against the complex loading of human tissue [1,3,4].

These smart wearable devices blend seamlessly into everyday clothing and accessories, paving the way for innovative applications for health and wellness monitoring. Wireless data collection is essential, and passive radio frequency identification (RFID) technology in the Ultra-High Frequency (UHF) band undoubtedly stands out as the ideal solution owing to its proven reliability and capability to harvest energy from the reader signals [5,6] and seamless integration with the Internet of Things [6].

RFID offers a battery-less architecture, harvesting energy directly from the interrogator’s signal, which facilitates long-term maintenance-free operation [5,6]. RFID systems operate on the principle of electromagnetic backscattering, employing tiny Integrated Circuit (IC) transponders that seamlessly integrate with low-profile, flexible wearable devices, capable of providing a read range of more than 10 m in the Ultra-High Frequency (UHF) range [7]. Additionally, modern RFID Integrated Circuits (ICs) have transitioned from simple ID transponders to sophisticated sensing nodes. They are capable of interfacing directly with epidermal sensors to monitor temperature, humidity, or biochemical markers, facilitating health monitoring by easily acquiring epidermal data through RFID tag-skin interaction without the need for additional complex signal-conditioning circuitry [2,8,9,10].

Despite these advantages, designing on-body RFID antennas presents a fundamental “antenna-in-environment” problem. The human body is a high-permittivity, lossy, and dispersive medium. When a standard antenna is placed in close proximity to the skin, the high dielectric constant of the underlying tissue causes severe impedance detuning, while the tissue conductivity leads to significant power absorption, resulting in the deterioration of antenna efficiency, read range, and thereby raising safety concerns [10,11,12,13,14]. This absorption is quantified by the Specific Absorption Rate (SAR), which must be minimized to comply with stringent safety standards (e.g., FCC/ICNIRP). Furthermore, the dielectric properties of the “platform” are not constant; they depend on the antenna operational frequency range and also vary significantly across different anatomical regions—such as the high-water-content muscle of the thigh versus the low-permittivity adipose tissue of the chest—and vary between individual patients [15,16,17,18,19]. This variability often necessitates a different antenna design for every body part, which is impractical for mass-market healthcare solutions.

To overcome these challenges and attain better on-body performance, conventional mitigation strategies have employed planar inverted-F antennas (PIFAs), microstrip patch antennas, and aperture antenna designs, relying on a metallic ground plane to isolate the radiator from the body [20,21,22,23]. However, at UHF frequencies, these structures typically require thick dielectric substrates or air gaps to maintain isolation and ensure sufficient bandwidth and radiation efficiency, leading to bulky, rigid profiles that hinder wearer comfort.

Traditional UHF RFID tag antennas employ a meandered dipole configuration, which provides long current paths, leading to miniaturized and low-cost operation [24,25,26,27]. However, these meandered tags experience destructive interference when placed on conductive platforms, such as the human body, as reflected signals combine out of phase with incident signals, reducing the read range [28]. Alternatively, patch antennas with full ground planes isolate underlying conductive objects, but suffer from a large size and low radiation efficiency [9,29].

One effective solution, offering a good height-efficiency trade-off, is to integrate the designed tag antenna with Artificial Magnetic Conductors, which have been proposed as high-impedance surfaces (HIS) [30,31,32,33]. Unlike a Perfect Electric Conductor (PEC) that reflects waves with a 180° phase shift—leading to destructive interference for low-profile antennas—an AMC acts as a “magnetic mirror.” These artificially engineered surfaces reflect, therefore, incident waves constructively with a near-zero phase shift within their resonance band, allowing the antenna to be placed extremely close to the surface without deteriorating its behavior [34,35,36,37]. In addition, the AMC structure also helps to suppress the back-lobe radiation by providing isolation from the underlying human body platform. This arrangement significantly enhances the antenna radiation gain and efficiency, reduces SAR (to comply with FCC standards), and improves overall on-body performance [34,35,36,37].

This intrinsic isolation property of AMCs is effectively utilized while integrating it with an arbitrary antenna, provided the antenna has smaller dimensions. The supported antenna may be of a wire type or a planar design. For optimum performance, appropriate spacing between the AMC and the antenna is often required. This gap can be conveniently filled through suitable dielectric support or lightweight materials, such as foam.

The gain enhancement due to isolation resulting from employing the AMC structure also depends upon the layout of the antenna. For instance, the Planar Inverted-F Antenna (PIFA) [38,39] with existing ground planes exhibits a gain improvement of a few decibels when integrated with AMC structures. Conversely, generic tag structures such as printed monopoles [40] and Yagi-Uda designs [41] exhibit a gain improvement exceeding 10 dB, highlighting the significant role of AMC structure support in delivering adequate radiation performance and realized gain under typical operating conditions.

Current literature has explored various AMC structures for on-body applications [32,33,34,35,36,42,43,44,45,46,47,48], aiming to achieve single-band [32,36,37,47,48] and multiband behavior [38,39,40,41,45,49,50]. However, most reported designs suffer from three critical shortcomings:

(i.) excessive footprints relative to the radiator size,
(ii.) performance degradation when subjected to mechanical bending (conformality),
(iii.) a lack of platform tolerance.

Many existing tags are optimized for a specific phantom and fail when moved to a body part with different dielectric properties [42,43,44,45,46,49,50], showing a weak permittivity invariance. Furthermore, miniaturizing AMC cells for the lower UHF RFID spectrum remains a significant design bottleneck due to the long wavelengths involved [24,25,28,29,37,47,48].

This research addresses these gaps by proposing a methodology for a novel, ultra-thin (2.23 mm) AMC-integrated RFID tag antenna for on-body biomedical applications. The proposed structure utilizes a thin (2 mm) silicon-doped flexible substrate for the AMC, and a 200 µm PET substrate for the tag, while all the metallic traces have been implemented using a 10 µm-thick silver-ink with conductivity 10^6^ S/m. The resulting 2.23 mm profile is significantly thinner than traditional AMC-backed designs, yet it provides high-gain and low-SAR operation, and the structure is fully planar, flexible, conformable, low-cost, and can be used as an epidermal antenna. The structure has been designed using CST Microwave Studio 2025, a general-purpose software for the 3D electromagnetic simulation of microwave components. CST is a well-assessed and established electromagnetic software for more than 20 years, as reported in the open literature for a wide range of applications (see, for example, [49,50,51,52,53,54]). The analysis presented in this work is based on high-fidelity numerical models developed in CST Studio Suite. While these results are predictive in nature, the underlying modeling methodology, electromagnetic solver settings, and material parameters have been rigorously grounded in a previously validated experimental framework. Specifically, the characterization of the flexible composite substrate and its complex electromagnetic interaction with human tissue has been verified through empirical measurements in [55], ensuring a high degree of reliability for the numerical predictions reported herein. Hence, we rely on CST results for assessing the performance of the proposed structure, ensuring the results align with established benchmarks for on-body epidermal applications.

Table 1 compares the proposed work with recent high-impact literature focusing on AMC-backed wearable antennas.

A critical analysis of the table above reveals several key advantages of the proposed methodology over existing State-of-the-Art designs:

Height and Profile (The “Zero-Gap” Advantage): Most existing designs (e.g., [42,43,44]) rely on significant air gaps or thick foam spacers ranging from 3 mm to 10 mm to achieve constructive reflection and decoupling. In contrast, the proposed structure achieves a record-low profile of 2.23 mm with zero air gap, directly integrating the tag with the AMC substrate without sacrificing performance.

Radiation Efficiency and Gain Increment: The gain improvement of 13 dB reported in this work is among the highest in the literature for the UHF band. Many textile-based antennas ([43,46]) show much lower increments, suggesting that their AMC structures are less efficient at suppressing back-lobe radiation into the lossy human body.

Electrical Compactness: With an electrical area of only 0.0246λ^2^, our antenna is significantly more compact than the majority of UWB and textile antennas (e.g., ref. [46] at 2.01 λ^2^). This miniaturization is achieved without the typical narrowband limitations associated with compact UHF designs.

Mechanical and Biological Compatibility: Unlike rigid FR4-based designs ([37,45]), our structure is fully flexible and conformable. Furthermore, whereas many designs ignore the impact of diverse phantoms, this work focuses on Robust Platform Tolerance, ensuring that the antenna does not require an air gap to maintain its resonance across different tissue types (fat, muscle, skin). While miniaturization is critical for implantable antennas—often restricted to less than 2% of the host body size [56]—epidermal sensors follow the ergonomics of medical patches and dressings. For these on-body applications, a length of 64.24 mm is standard for clinical use, comparable to commercial ECG or wound-care patches. The critical factor for wearer comfort at this scale is not the footprint itself, but the mechanical conformability and flexibility, which are addressed here by the reduced thickness and the silicone-titanate substrate. Frequency Target: While many recent works focus on the 2.45 GHz or 5.8 GHz ISM bands ([42,43,44,46])—where antenna dimensions are naturally smaller—this work tackles the more challenging 868 MHz UHF RFID band, delivering a compact and ultra-thin solution where wavelength-related size constraints are much more severe.

The performance of the proposed RFID tag was rigorously evaluated and compared under two distinct operating conditions: directly mounted on the human body and integrated with a custom-designed Artificial Magnetic Conductor (AMC) structure. The AMC layer serves as an electromagnetic shield, effectively isolating the tag from the high-loss dielectric environment of the wearer’s body. Experimental results demonstrate a substantial enhancement in the tag antenna gain, yielding an increase of approximately 13 dB. This gain improvement—nearly an order of magnitude—significantly outperforms typical increments reported for similar wearable devices in the literature (as shown in Table 1). Consequently, this isolation leads to a marked extension of the read range, enabling reliable performance in standard operating scenarios and establishing the platform as a robust, “platform-tolerant” solution. Earlier studies on miniaturized biomedical antennas, such as the meander-line structures reported in [56], achieve extreme compactness (6 × 6 mm^2^) suitable for implants. However, these designs suffer from significant gain suppression (approx. −21.8 dBi) and high SAR, due to the absence of a shielding layer. In contrast, the proposed AMC-backed tag, while having a larger footprint (32 × 64 mm^2^), provides a gain enhancement of more than 13 dB compared to unshielded designs on the body. This trade-off is essential for epidermal applications where long-range communication is required. When compared to similar AMC-based on-body antennas (Table 1), the proposed design maintains a lower or comparable profile and area and high radiative efficiency.

The designed antenna exhibits very good robustness and high reliability, maintaining stable performance regardless of the proximity to the human body and of bending conditions. This stability is largely attributed to the optimized isolation provided by the AMC planar structure. The proposed structure differentiates itself from prior AMC-backed RFID designs through its ‘zero-gap’ integration, achieving an ultra-low profile of 2.23 mm without the need for air spacers or thick dielectric buffers commonly required to maintain resonance. Furthermore, while meander-line structures (e.g., [56]) offer extreme miniaturization for implantable use, they suffer from significant gain suppression (down to −21.8 dBi). The design presented here prioritizes radiative efficiency for epidermal sensing, providing a 24 dB gain advantage over miniaturized implantable alternatives while maintaining a highly compact footprint. Moreover, the implementation utilizes ultra-thin dielectric substrates for both the AMC and the RFID tag. By tailoring the AMC substrate to combine a high dielectric constant with low tangential losses, we have achieved a configuration that is simultaneously flexible and biocompatible. Due to these specific material properties, the RFID tag is uniquely suited for use as an epidermal antenna. This configuration supports high-fidelity “on-skin” sampling of critical physiological and environmental parameters, including: surface and core body temperature, pH levels and humidity, skin impedance and electrophysiological potentials, mechanical deformations and fluid exchanges at the skin interface. This versatility positions the device as a high-performance tool for real-time health monitoring and advanced wearable sensing applications.

## 2. Design of the AMC Unit Cell

**Figure 1:**
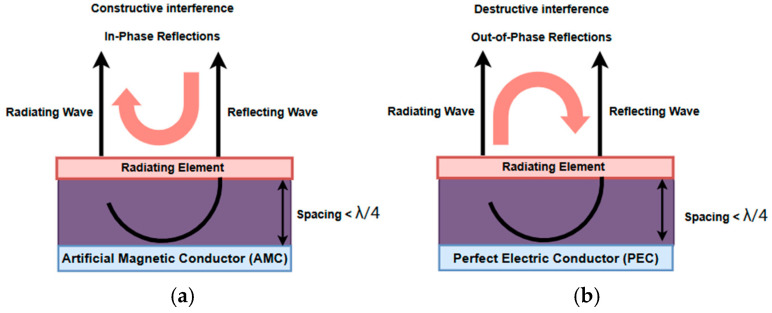
EM Wave transmission on Reflector (**a**) PEC and (**b**)AMC structures [6].

Artificial Magnetic Conductors (AMCs), often categorized as High-Impedance Surfaces (HIS), are engineered metasurfaces acting as periodic structures, designed to manipulate the propagation and reflection of incident electromagnetic (EM) waves under different incident angles or polarization conditions. These metasurface structures are designed to resonate and exhibit filtering characteristics, thereby benefiting various microwave applications through their compact size and high gain. Unlike a conventional Perfect Electric Conductor (PEC), which exhibits a 180° reflection phase (causing destructive interference and signal cancellation when a radiating element is placed in close proximity), an AMC acts as a “magnetic mirror”, as demonstrated in Figure 1. It reflects incident waves with a nearly zero-degree phase shift at its resonant frequency.

The operational principle of an AMC relies on the resonance occurring within the effective cavity formed between the periodic top metallization and the continuous ground plane. This unique property allows for the design of ultra-low-profile antennas, as the radiating element can be placed directly on the metasurface layer, which represents the AMC surface, without impedance detuning or gain degradation. For wearable applications, antenna performance deteriorates when placed directly on the human body due to its high dielectric properties, resulting in impedance detuning and reduced efficiency and gain. Thus, to attain isolation and robust performance, AMC can be placed between the antenna and the human body platform, since it serves as an effective isolation shield, decoupling the antenna from the high-permittivity, lossy tissues of the human body. AMC structure particularly helps enhance the radiation properties, and thus the gain compared to the antenna affixed directly to the human body. Additionally, these structures demonstrate varied applications such as acting as resonant structures, acting as a ground layer of an antenna, isolating or shielding the radiating structures, improving the radiation performance in wearable antennas by enhancing radiation efficiency and stabilizing gain, lowering the Specific Absorption Rate (SAR) levels ensuring safety compliance for long-term monitoring, and enabling detection of antennas and their functioning in tunable devices.

**Figure 2:**
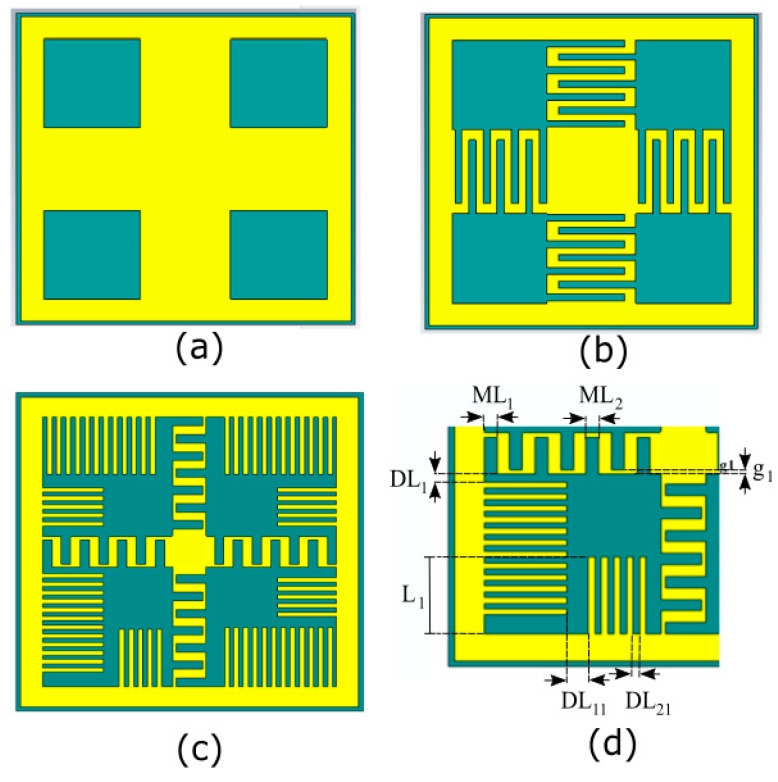
AMC Unit Cell Structure Design Evolution: (**a**) Square Patch; (**b**) Meandered Cross; (**c**) Final Interdigitated Comb Structure; (**d**) Main geometrical parameters. DL_1_ = 1.06 mm; DL_11_ = 1.3 mm; DL_21_ = 0.43 mm; ML_1_ = 0.87 mm; ML_2_ = 0.87 mm; L_1_ = 5.78 mm; g_1_ = 0.30 mm.

The primary challenge in UHF-RFID designs (865–928 MHz) is the physical size of the metasurface, as conventional unit cells are often too bulky for on-body and epidermal applications [55,56]. To achieve a compact footprint, this work employs a structured design evolution (see Figure 2a–d):

1. The design begins with a symmetric square patch inspired by the “Jerusalem Cross” geometry. This establishes the baseline High-Impedance Surface (HIS) characteristics. The structure can be modeled as a parallel LC resonant circuit, where the gaps between adjacent patches provide the capacitance C, and the narrow microstrip segments provide the inductance L.
2. To shift the resonance to the lower UHF band without increasing the physical area, the arms of the cross are meandered. This increases the electrical length of the surface current paths, effectively introducing additional inductive reactance.
3. Further miniaturization is achieved by incorporating multiple parallel strips (comb-like arms) in each quadrant. These function as interdigitated slots, significantly boosting the coupling capacitance between unit cells. This high-density capacitive loading allows the modified AMC to resonate at a much lower frequency than a standard patch of the same dimensions.
4. The structure is fine-tuned by varying the inter-cell spacing (D_Lcell_), the lengths of the comb-arms (L_1_, L_2_, L_3_), and the overall unit cell periodicity to lock the 0° reflection phase precisely at the center frequency of 866 MHz.

A primary challenge in the design and implementation of AMC structures for UHF applications is achieving a compact physical footprint [57,58]. While optimizing the periodic geometry is essential, the precise engineering and fabrication of a high-permittivity substrate are equally critical. Given the inherently narrowband characteristics of AMC structures, the substrate dielectric properties must be meticulously tailored; even marginal deviations can significantly degrade device performance or shift the operational frequency out of specification. To realize a substrate meeting these stringent requirements, we exploit the work described in [59], which developed a nanocomposite material by embedding a highly ferroelectric ceramic filler within a silicone rubber matrix. An important innovation of this work also relies on the use of an in-house-made, high-permittivity, flexible, and biocompatible dielectric substrate, which allows balancing the requirements of miniaturization and wearer comfort. Silicone rubber was selected as the composite matrix because it is biocompatible, non-toxic, and easy to process, while remaining sufficiently fluid during pre-curing to allow uniform filler dispersion and molding. Careful mixing, preferably with air-removal techniques, is required to avoid microbubbles that would degrade the dielectric performance. Barium Titanate (BaTiO_3_) was chosen as the ceramic filler for its high dielectric constant, which is strongly dependent on its crystalline phase. Since BaTiO_3_ undergoes a phase transition above about 120 °C that significantly reduces permittivity, all processing is carried out at room temperature to preserve a stable dielectric constant of approximately 250. To predict the effective permittivity ε_eff_ of the final composite, the Lichtenecker logarithmic mixture model [60] was applied:

ln(ε_eff_) = f_1_ × ln (ε_1_) + f_2_ × ln (ε_2_) (1)

where the following applies:

- ε_eff_ is the effective dielectric constant of the composite.
- ε_1_ and ε_2_ are the dielectric constants of the matrix (3.25) and the filler (250), respectively.
- f_1_ and f_2_ represent the respective volume fractions of the materials.

For this AMC design, substrates with an ε_eff_ of 6.24 and a loss tangent of 0.0135 were selected. According to the model, this can be achieved using a filler volume fraction of approximately 16%. The use of this high-permittivity substrate further shrinks the guided wavelength λ_g_, allowing the AMC unit cell to reach a miniaturized state that is ideal for discrete, on-body RFID tags. The conductive layers were modeled with a 10 μm thickness. Given the ink conductivity (10^6^ S/m), this value is comparable to the skin depth (17.1 μm at 866 MHz), representing a practical compromise between fabrication constraints, mechanical flexibility for wearable use, and electromagnetic efficiency.

The final AMC design has a unit cell with periodicity D = 32.12 mm (0.11 λ_0_ at 868 MHz) and a total thickness of 2.02 mm (0.007 λ_0_ at 868 MHz). The conventional AMC cell with the same geometry will resonate beyond 1.75 GHz; therefore, the comb-like arms allow a size reduction of almost 50%.

**Figure 3:**
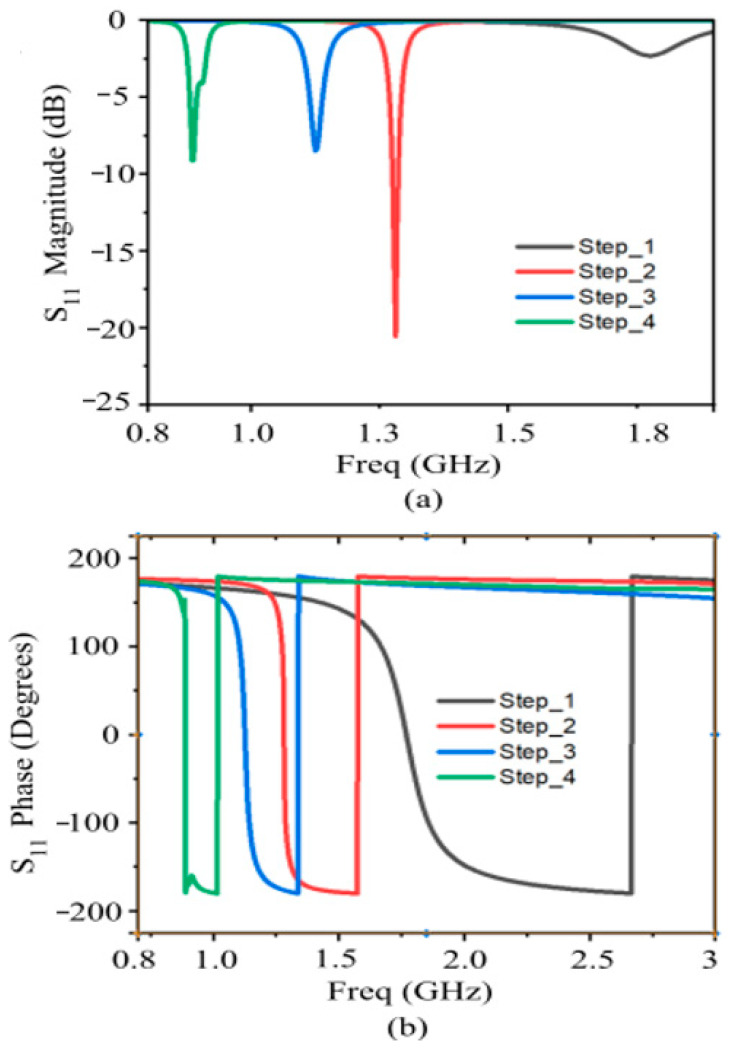
Frequency response of the proposed AMC cell for the four evolution steps: (**a**) Reflection coefficient magnitude and (**b**) phase. The final design achieves a +12° phase at 866 MHz with a 30 MHz ± 90° bandwidth (850–880 MHz).

**Figure 4:**
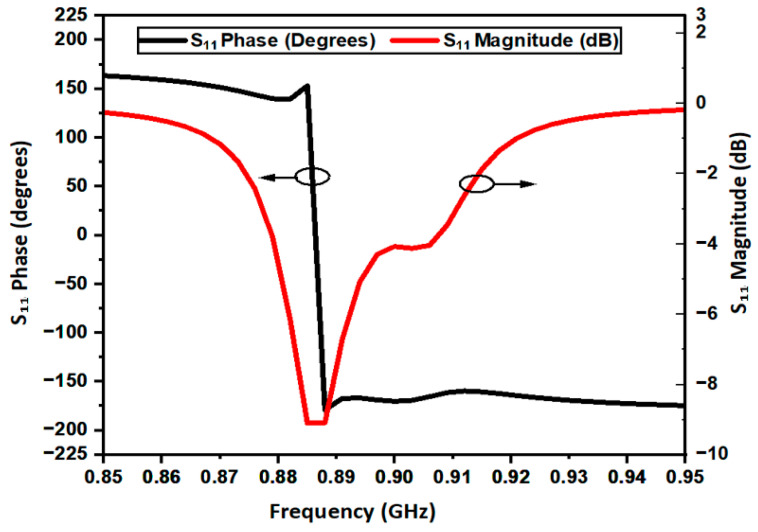
Frequency response of the final optimized AMC (fourth evolution step). AMC operating point at 866 MHz: +12° reflection phase, −35 dB S_11_ magnitude, confirming quasi-PMC behavior within the ETSI RFID band.

**Figure 5:**
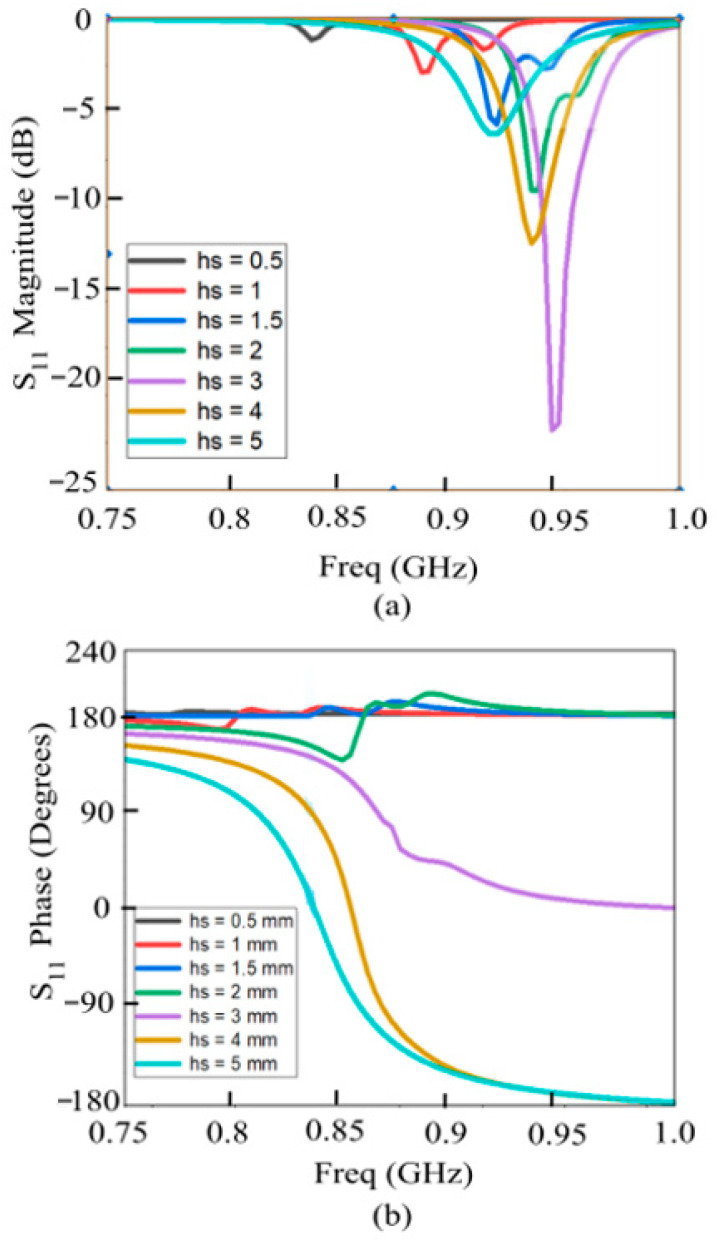
Frequency response of the proposed AMC cell for different substrate thicknesses: (**a**) Reflection coefficient magnitude and (**b**) phase.

The reflection coefficient (amplitude and phase) of the AMC structure across the four evolution steps is reported in Figure 3a,b, while Figure 4 illustrates the performance of the final optimized configuration. Furthermore, Figure 5a,b highlights the impact of substrate thickness ‘h_s_’ on the AMC response. Increasing the thickness leads to a broader operating bandwidth and a downward shift in the resonant frequency. Notably, for thicknesses below 3 mm, the AMC exhibits only a partial phase inversion. While thicker substrates would improve bandwidth, epidermal and on-body applications demand a low-profile, comfortable, and unobtrusive design. Consequently, to balance electromagnetic performance with wearable constraints, the substrate thickness was fixed at 2 mm. Although these results show only a partial phase inversion (as seen also in Figure 2 and Figure 3), it remains sufficient to ensure satisfactory performance when the integrated RFID tag is deployed on the human body, as demonstrated by the results presented in the following sections. The electromagnetic effectiveness of the AMC is determined by its reflection phase characteristics. In this work, the AMC operational bandwidth is defined by the frequency range where the phase remains within ±90°, identifying the region of constructive interference. At the center frequency of 866 MHz, the unit cell exhibits a reflection phase of +12°, confirming near-ideal quasi-PMC behavior. The resulting ±90° bandwidth is 30 MHz (850–880 MHz), which provides a sufficient safety margin to cover the entire ETSI RFID band (865–868 MHz) even under the influence of body-loading effects.

## 3. Proposed RFID Tag

**Figure 6:**
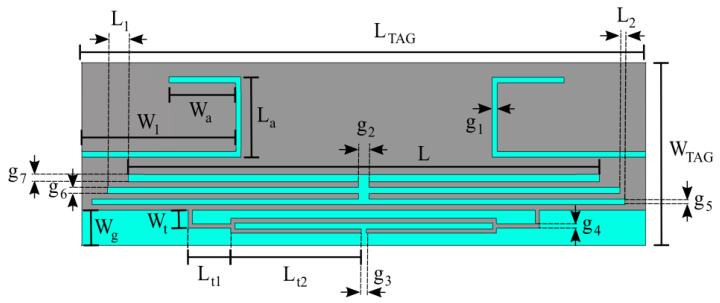
Proposed multi-strip slotted RFID tag antenna Design. L_TAG_ = 59.43 mm; W_TAG_ = 17.29 mm; L = 49.71 mm; W_1_ = 16.21 mm; W_a_ = 7.56 mm; L_a_ = 7.56 mm; g_1_ = 0.54 mm; g_2_ = 1.08 mm; g_3_ = 0.5 mm; g_4_ = 0.4 mm; g_5_ = 0.54 mm; g_6_ = 0.54 mm; g_7_ = 0.67 mm; L_1_ = 2.16 mm; L_2_ = 1.62 mm; L_t1_ = 4.5 mm; L_t2_ = 13.75 mm; W_g_ = 3.35 mm; W_t_ = 1.7 mm.

This section discusses design and optimization of the proposed RFID tag antenna (whose final layout is shown in Figure 6), which is focused on the European UHF-RFID band (865–870 MHz), with a target center frequency of 866 MHz. The antenna is realized by using a 10 µm thick silver ink for the metal traces, printed onto a low-cost PET substrate. The substrate has a thickness of 0.2 mm, a relative permittivity of 3, and a loss tangent of 0.04. The final optimized physical footprint of the antenna is a compact 59.43 × 17.29 × 0.2 mm^3^. The performance of a passive RFID tag is primarily governed by the power transfer efficiency between the antenna and the Integrated Circuit (IC). For this design, the Impinj Monza 4 chip was selected for connection to the tag feed terminal (g_3_ in Figure 6), offering a high sensitivity of −20 dBm. To ensure maximum power transfer from the tag antenna to the employed chip, the antenna impedance (Z_ant_ = R_ant_ + jX_ant_) must be the complex conjugate of the chip impedance (*Z_chip_* = R_chip_ + jX_chip_). The impedance of the RFID chip is determined from the information provided in its datasheet [61]. The chip’s electrical characteristics are typically provided as a parallel RC network, and for the selected chip, we have *R_p_* = 1.65 kΩ and *C_p_* = 1.21 pF. To facilitate the design of the matching network, these values are converted into an equivalent series impedance at 866 MHz using Equation (2) below:

(2) $$Z_{chip}=\;\frac{R_{p}}{1+\omega^{2}R_{p}^{2}C_{p}^{2}}-\;j\;\frac{\omega R_{p}^{2}C_{p}}{1+\omega^{2}R_{p}^{2}C_{p}^{2}}\;\approx \;13\;-\;j\;151\;Ω$$

Consequently, the antenna must be designed to exhibit a highly inductive input impedance of approximately 13 + j 151 Ω to achieve perfect conjugate matching with the impedance of the employed Impinj Monza 4chip and maximize the read range.

Achieving a high inductive reactance in a compact form factor is addressed through two primary design features:

- A T-match structure is integrated at the feed terminal (g_3_ in Figure 6). This network acts as an impedance transformer, allowing for the precise tuning of the inductive reactance X_ant_ to cancel the chip capacitive reactance. By adjusting the dimensions of the T-match arms and their proximity to the main radiator, the resistance R_ant_ is also transformed to match the low 13 Ω requirement of the chip.
- The radiating body employs a modified patch structure incorporating two symmetrically positioned U-shaped slots. These slots serve a dual purpose:
  ○ They force the surface currents to meander, effectively increasing the electrical length of the antenna without increasing its physical size. This enables the antenna to resonate at 866 MHz despite its sub-wavelength dimensions, facilitating miniaturization and achieving a compact physical footprint.
  ○ The U-shaped geometry introduces additional distributed capacitive and inductive loadings, which influence the overall electrical length of the antenna and its resonant behavior. This provides the design flexibility needed to fine-tune the resonance and broaden the Impedance Bandwidth, ensuring the tag remains functional despite the frequency shifts often caused by varying environmental conditions or proximity to diverse materials.

**Figure 7:**
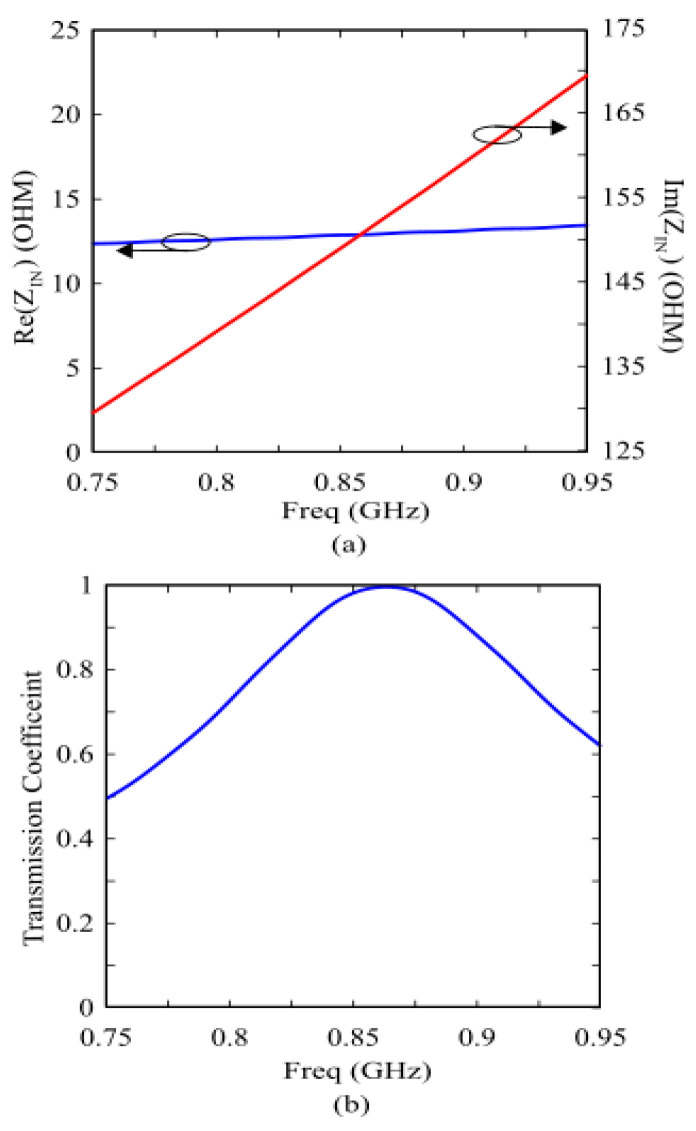
Frequency response of the designed TAG. (**a**) Input impedance (R_in_, X_in_). (**b**) Transmission coefficient (τ).

Furthermore, to enhance design flexibility and fine-tune the impedance, multi-strip slots are integrated, which further broaden the impedance bandwidth, ensuring robust performance of RFID systems across diverse environmental conditions. The dimensions of the central radiating section and the stubs at each end were optimized through parametric sweeps in CST Studio Suite. These “comb-like” multi-strip slots integrate additional degrees of freedom, allowing for independent control over the center frequency and the impedance bandwidth. This robust design ensures that the antenna maintains its performance characteristics when transitioned from a free-space environment to being integrated with the AMC shielding layer described in the subsequent section. Figure 7 illustrates the simulated input impedance and the power transmission coefficient *τ* of the designed tag in free space. The coefficient *τ* accounts for the impedance mismatch between the antenna and the microchip, and is defined as Equation (3) below:

(3) $$\tau =\frac{{4R}_{in}R_{chip}}{{\left(R_{in}+R_{chip}\right)}^{2}+{\left(X_{in}+X_{chip}\right)}^{2}}$$

where Z_in_ = *R_in_* + jX_in_ and Z_chip_ = *R_chip_* + jX_chip_ represent the complex input impedances of the tag antenna and the RFID chip, respectively. The results demonstrate excellent performance within the European UHF RFID band (865–870 MHz). Furthermore, the transmission coefficient bandwidth, defined as the frequency range where *τ* > 0.8, extends from 820 MHz to 930 MHz. This broad operational range ensures full compliance not only with European regulations but also with the US UHF RFID band (902–928 MHz).

### Numerical Methodology and Methodology Validation

To ensure the accuracy of the numerical analysis, the simulation environment utilizes material parameters derived from experimental characterization reported in previous work [55]. The flexible substrate consists of a silicone rubber matrix doped with Barium Titanate (BaTiO_3_) ceramic filler at a 16% volume fraction. This specific composite was previously fabricated and tested, yielding a measured dielectric constant εr of 6.24 and a loss tangent tanδ of 0.0135 at 866 MHz. Furthermore, the modeling approach for the AMC unit cell and its on-body performance has been validated against experimental data obtained from physical phantoms and human volunteers for similar structures [55]. The agreement between the measured reflection phase and the simulated quasi-PMC (Perfect Magnetic Conductor) bandwidth in those studies confirms that the current numerical setup accurately captures the physics of the antenna-body coupling and the shielding effectiveness of the metasurface.

## 4. Numerical Results and On-Body Performance

To evaluate the robustness and efficiency of the proposed RFID system, its performance was analyzed using a high-fidelity numerical human body phantom within the CST Studio Suite environment. This section compares two distinct operational scenarios to highlight the performance enhancement provided by the AMC shielding structure: the standalone RFID tag placed directly on the skin, and the integrated RFID-AMC system (tag mounted on the tailored AMC structure) attached to the phantom.

### 4.1. Human Body Phantom and Tissue Modeling

**Figure 8:**
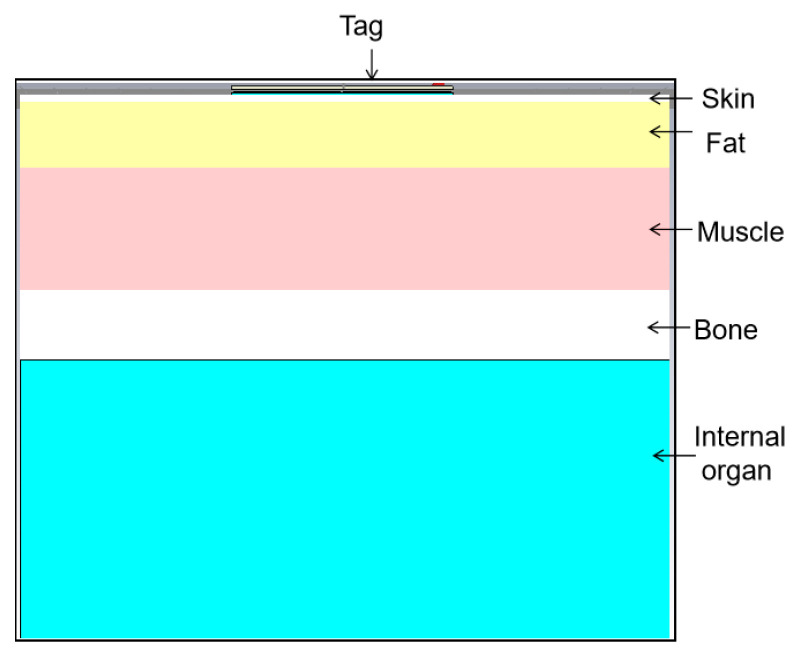
Simplified multilayer human body model.

To realistically simulate the electromagnetic interaction between the antenna and biological tissues, a multilayer heterogeneous human body model (with an area of 200 × 200 mm^2^) was employed. The phantom accurately replicates the human anatomy through five distinct layers: Skin, Fat, Muscle, Bone, and Internal Organ, as illustrated in Figure 8. The specific electromagnetic properties (permittivity εr and conductivity σ) and layer thicknesses at the center frequency of 866 MHz are summarized in Table 2. The phantom dimensions (200 × 200 mm^2^, approximately 0.6 λ at 868 MHz) were chosen to represent a localized anatomical region, such as the arm or chest. To mitigate potential diffraction effects from the phantom edges, Perfectly Matched Layer (PML) boundary conditions were applied at the simulation boundaries. This setting ensures that outgoing radiation is absorbed without unphysical reflections back into the computational domain, providing an accurate estimation of the far-field metrics and radiation patterns in an on-body environment.

### 4.2. Comparison of Scenarios: Standalone vs. AMC-Integrated Tag

- Scenario I: RFID Tag Directly on Phantom

**Figure 9:**
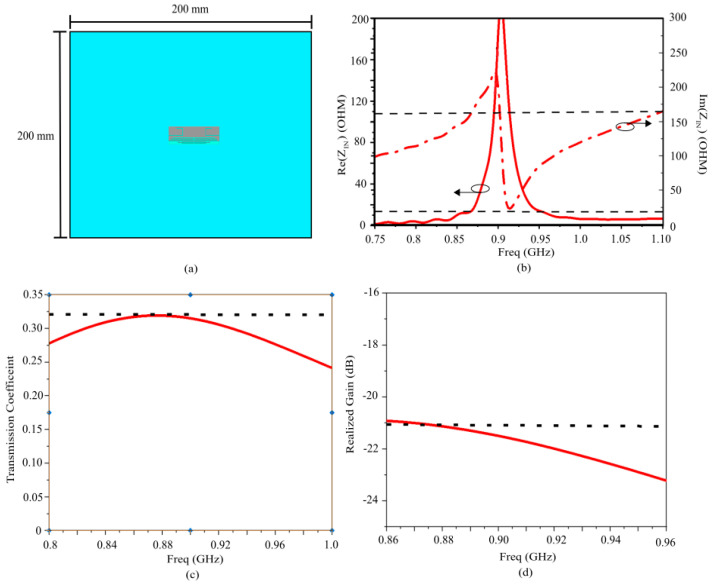
Results for the designed TAG directly attached to the phantom. (**a**) Top view; (**b**) TAG Input Impedance; (**c**) TAG transmission coefficient; (**d**) TAG realized gain.

When the RFID tag is placed directly on the skin without a backing structure, the high permittivity and conductivity of the body lead to severe impedance mismatch and high absorption losses. Figure 9 displays the simulated input impedance, transmission coefficient, and realized gain. The dissipative nature of the tissues effectively “shorts” the antenna, resulting in a very low realized gain of only −21 dB at 868 MHz, a transmission coefficient below 0.32, and a consequently reduced read range of less than 0.5 m.

- Scenario II: RFID Tag Integrated with 2 × 1 AMC Array

In the second scenario, the tag is integrated with the designed AMC metasurface array, consisting of 2 × 1 unit cells, which represents the smallest possible size for a finite AMC.

**Figure 10:**
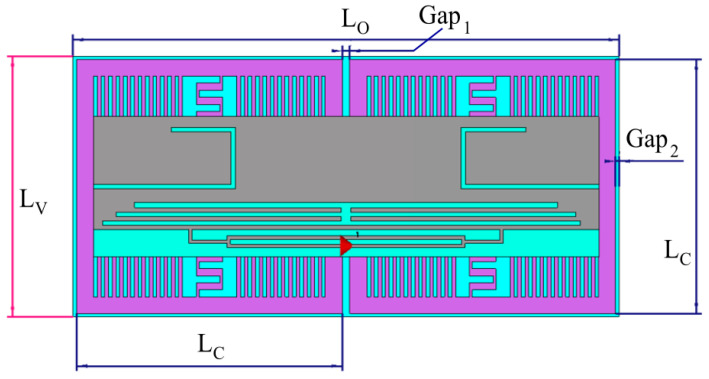
Designed AMC-Backed RFID Tag Antenna. L_O_ = 64.24 mm; L_V_ = 32.12 mm; L_C_ = 31.25 mm; Gap_1_ = 0.87 mm; Gap_2_ = 0.43 mm.

The tag is placed directly upon the AMC without any separation air gap (Figure 10). The integrated structure maintains an ultra-compact footprint of 64.24 × 32.12 × 2 mm^3^ (0.017 λ_0_^3^), achieving significant miniaturization for an on-body tag antenna at the operating frequency of 866 MHz.

The AMC acts as a high-impedance surface, approximating a “perfect magnetic conductor” (PMC) condition. This prevents the electromagnetic fields from penetrating the lossy body tissues. The inclusion of the AMC leads to a considerable gain increase of 13 dB compared to the standalone case, while maintaining a high front-to-back ratio and low cross-polarization levels. This electromagnetic isolation significantly enhances the read range, making the tag suitable for long-range biomedical monitoring.

**Figure 11:**
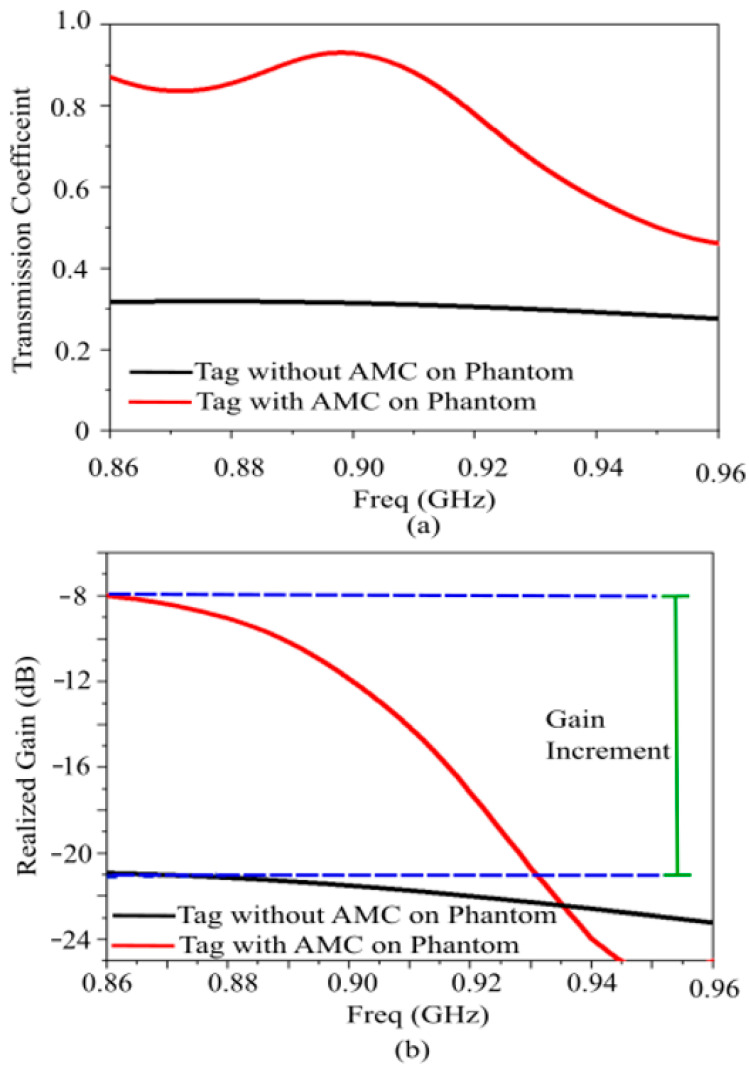
Results for the designed TAG with AMC on the phantom. (**a**) Transmission coefficient. (**b**) Realized gain.

Figure 11 compares the simulated transmission coefficient and realized gain for both scenarios. The power transmission coefficient remains stable within the 865–870 MHz band and extends up to 920 MHz, even in the presence of the phantom. This indicates that the AMC provides an effective buffer that preserves the conjugate matching between the IC and the antenna.

To further validate the unique contribution of the Artificial Magnetic Conductor, the proposed tag was also evaluated against a conventional Perfect Electric Conductor (PEC) ground plane of the same footprint. Numerical results show that a metallic isolation layer is ineffective at this thickness: due to the 180° phase reversal of image currents, the antenna gain drops to less than −26 dBi at 868 MHz. This confirms that the AMC’s ability to provide a 0° reflection phase is the critical mechanism enabling constructive interference and the high radiative efficiency required for on-body communication.

A 2 × 1 AMC array configuration was selected to achieve a balance between electrical performance and the small physical footprint (0.0246 λ^2^) required for epidermal comfort. While finite arrays are subject to edge effects, the 2 × 1 arrangement is sufficient to establish the High Impedance Surface (HIS) conditions necessary for isolation. This is validated by the 13 dB gain improvement and the significant reduction in SAR compared to the standalone tag, demonstrating that even a minimal array provides effective shielding from the lossy human tissue.

**Figure 12:**
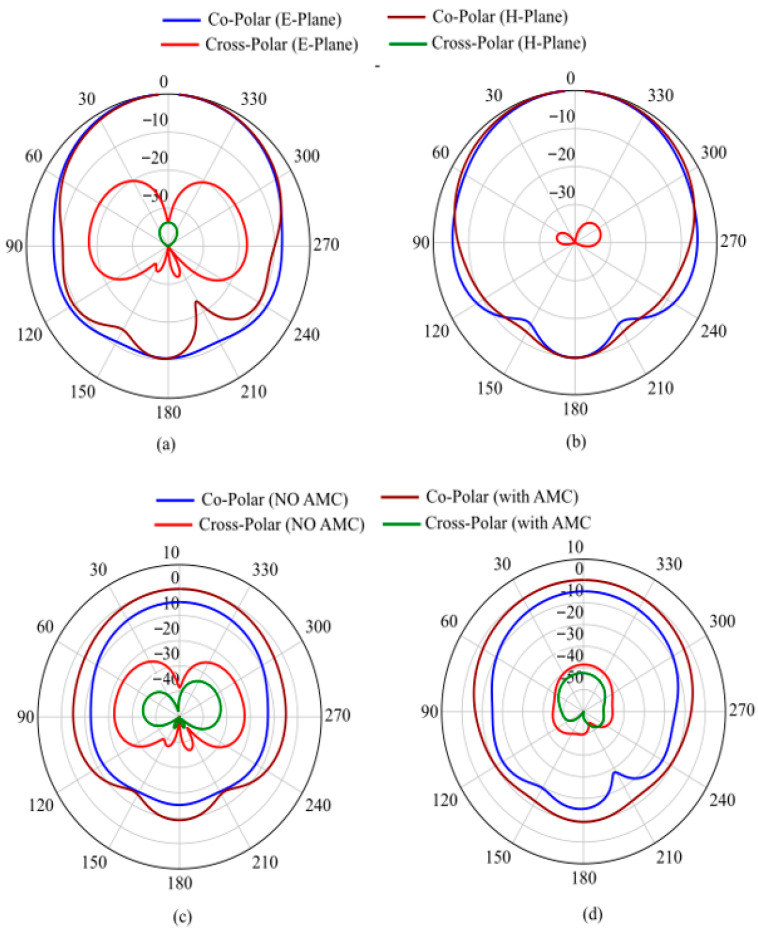
Normalized far field pattern (**a**) when the tag is directly attached to the phantom and (**b**) when the tag is placed upon the tailored AMC structure. Comparison between the Far Field Patterns: (**c**) E-Plane; (**d**) H-Plane.

**Figure 13:**
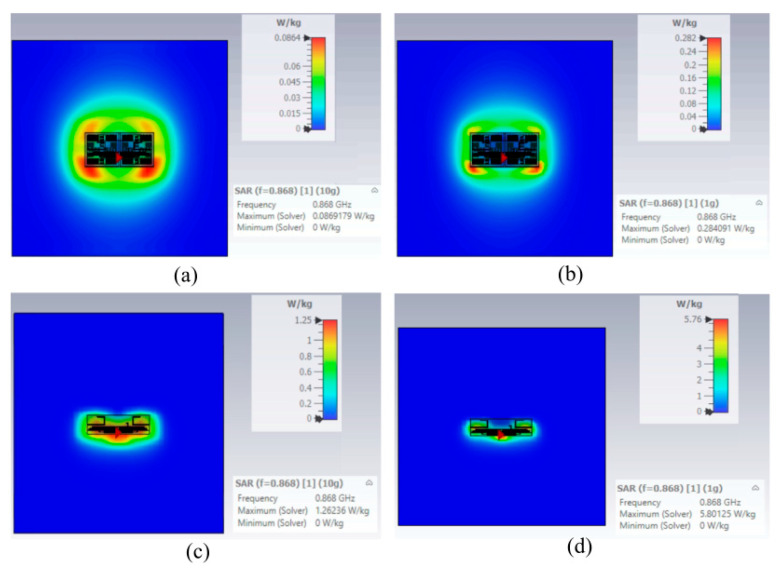
SAR values when the tag is placed upon the tailored AMC structure for (**a**) 10 g and (**b**) 1 g tissue masses. SAR values when the tag is directly attached to the phantom for (**c**) 10 g and (**d**) 1 g tissue masses.

The presented results confirm that the AMC structure serves as an effective shielding layer between the antenna and the human body. This ensures system robustness against the ‘body-loading’ effect typical of wearable electronics while providing high isolation. This behavior is further validated by the far-field patterns in Figure 12: the AMC-backed tag exhibits a cross-polar component significantly lower than the standalone configuration (with a 10 dB reduction in peak values) and a front-to-back ratio exceeding 10 dB. Furthermore, the SAR values for 10 g and 1 g tissue masses, as illustrated in Figure 13, demonstrate that the AMC-shielded structure reduces SAR by more than an order of magnitude compared to the standalone tag. The attained SAR values for both scenarios are summarized in Table 3.

As shown in Figure 11b, the 13 dB gain increment allowed by the AMC represents a significant improvement for the performance of the proposed on-body/epidermal device, enabling reliable long-range communication. This shielding benefit is inherent to the AMC design and can be effectively applied to various antenna geometries, provided the antenna footprint remains within the AMC boundaries. As evidenced by the State-of-the-Art comparison in Table 1, the AMC functions as a high-performance, independent isolation layer, with the specific gain and isolation levels determined by the synergy between the AMC layout and the supported antenna.

### 4.3. RFID System Performance

To evaluate the system link budget in a manner consistent with clinical requirements [62], the maximum read range r was calculated. For a passive UHF RFID system, r defines the limit of the link margin. Using the Friis transmission equation with a 3.28 W *EIRP* at the reader (standard for 2 W ERP), a chip sensitivity *P_th_* of −18 dBm, and the simulated on-body realized gain of −8 dBi at 868 MHz, the tag achieves a theoretical on-body range of 5 m:

(4) $$R\;=\;\frac{\lambda }{4\pi }\sqrt{\frac{EIRP\;\cdot \;G_{r}\;\cdot \;{ƞ}_{p}}{P_{th}}}$$

where the following applies:

- r is the maximum read range
- λ is the wavelength at the operating frequency
- EIRP is the equivalent isotropically radiated power of the reader
- $G_{R}$ is the realized gain of the tag antenna ($G_{R}=G_{tag}\tau$), which accounts for both the antenna gain and the impedance matching between the antenna and the RFID chip
- $P_{th}$ is the minimum power required to activate the chip (chip sensitivity)

In real-world clinical scenarios, considering a polarization mismatch factor between reader and tag antennas, pm = 0.5, a robust read range of over 3.6 m is guaranteed. This confirms that the AMC effectively restores the link margin lost to body-tissue absorption, which typically renders unshielded tags unreadable at distances greater than a few centimeters.

## 5. Robustness and Bending Validation

The practical utility of a wearable RFID tag depends heavily on its ability to maintain stable performance despite environmental and mechanical variations. This section rigorously assesses the sensitivity and robustness of the proposed on-body AMC-Backed RFID Tag Antenna under mechanical deformation (bending). In wearable applications, tags are frequently subjected to non-planar mounting on limbs, such as arms or legs. To validate performance in these dynamic conditions, the system was simulated on a cylindrical phantom with varying radii, representing different anatomical dimensions.

**Figure 14:**
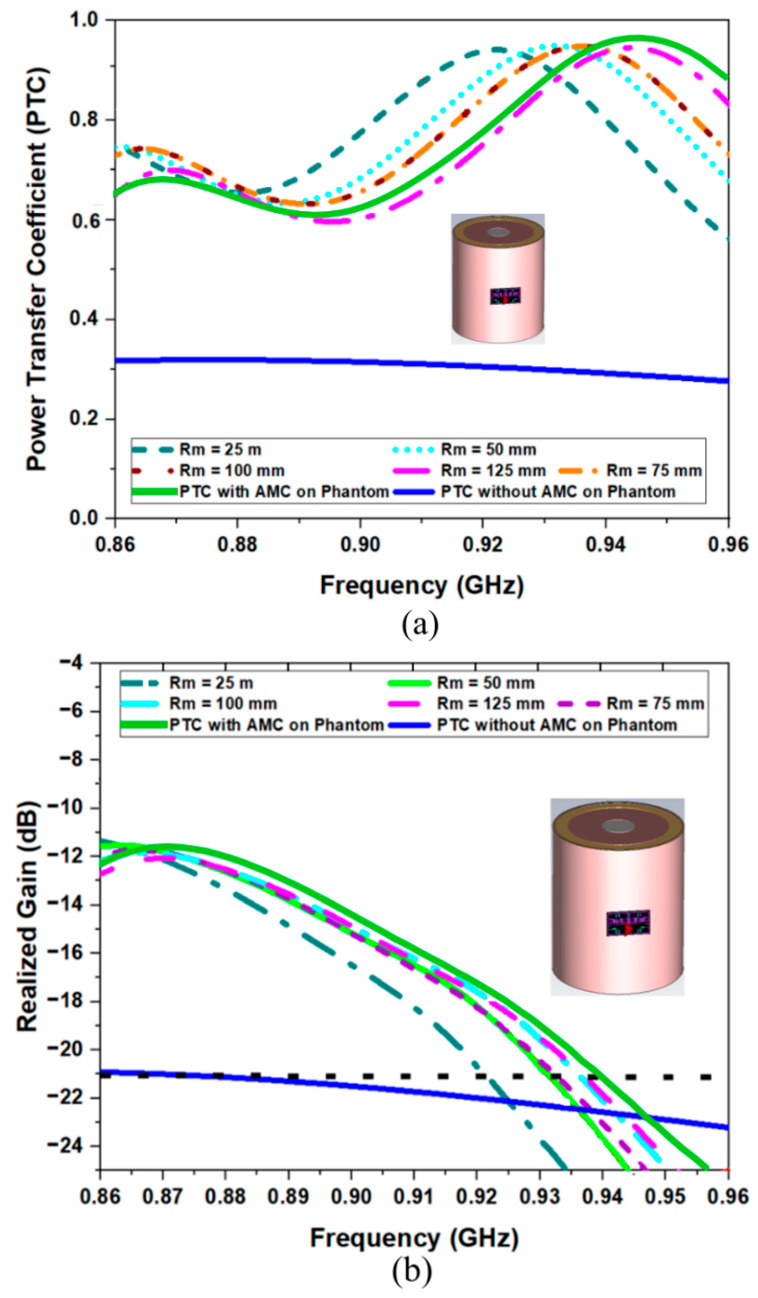
(**a**) Simulated Power Transmission Coefficient and (**b**) Realized Gain for the proposed AMC-Backed RFID Tag Antenna attached to the multilayer leg model for different radii R_m_ of muscle layer, and hence for different bending diameters. The continuous green curve corresponds to the planar reference with a metal thickness of the tag equal to 80 μm.

Figure 14 illustrates the simulated Power Transmission Coefficient and Realized Gain for the AMC-backed tag attached to a multilayer leg model across different radii (Rm) of the muscle layer, and hence different bending diameters. By varying the thickness of the muscle layer and, consequently, the overall phantom diameter, we explored different antenna bending diameters ranging from 150 mm to 300 mm. This parametric exploration also enabled us to evaluate antenna sensitivity within a multilayer cylindrical phantom model. Despite the substantial variations in layer thickness, the antenna exhibited remarkable stability in performance. As shown in Figure 14b, both the realized gain and the transmission coefficient curves remain highly consistent regardless of the phantom radius within the designated operating frequency band (865–870 MHz). This resilience can be attributed to the high isolation provided by the supporting AMC structure. It highlights the platform tolerance of our structure, showing its low sensitivity and excellent robustness regarding both the degree of bending and the specific dimensions of the underlying multilayer phantom.

To ensure numerical stability and prevent mesh clashing or self-intersection errors during bending in CST Microwave Studio, the metal thickness in the bent configuration was set to 80 μm. While the planar model uses a 10 μm thickness, which accurately represents the thin-film application, the skin depth of the silver ink (σ = 10^6^ S/m) is approximately 17 μm at 868 MHz. By increasing the thickness to 80 μm in the bent model, the simulation actually slightly underestimates the conductor losses compared to the 10 μm physical reality. However, this adjustment was necessary for mesh convergence. The resulting gain stability observed in simulations, therefore, serves as a conservative benchmark, confirming that the electromagnetic performance remains robust despite the geometric deformation. This modification strongly alters the volume of the conductive material, thereby modifying resistive losses and surface current distribution. Consequently, the observed gain reduction is a numerical artifact; the physical performance is expected to remain even more stable than the numerical model suggests.

The most significant finding of this analysis is the stability of the electrical response relative to the bending radius. The performance metrics (Gain and Power Transmission) remain virtually identical whether the tag is mounted on a small-radius limb (e.g., a wrist) or a larger-radius limb (e.g., a thigh). This high level of robustness validates two key system attributes:

- Mechanical Resilience: The proposed layout is electrically stable. Variations in curvature (simulating body movement or diverse mounting surfaces) do not significantly perturb antenna tuning or radiation efficiency.
- Operational Integrity: The low sensitivity to bending ensures that communication with the RFID reader is maintained even when the device is worn over joints or utilized in highly dynamic, non-static environments. This makes it an ideal candidate for long-term health monitoring and epidermal sensing.

### Limitations and Future Work

While the numerical results demonstrate the superior performance of the AMC-backed tag, it is important to acknowledge the inherent limitations of a purely computational study. Real-world performance may be influenced by fabrication tolerances, such as the precision of the silver nanoparticle screen-printing process, variations in the cured ink thickness, and the uniformity of the BaTiO_3_ filler dispersion within the silicone matrix. Additionally, mechanical deformations during extreme bending may introduce minor shifts in the operational frequency not fully captured by static models. Future research will focus on the physical prototyping of this miniaturized geometry to experimentally quantify these effects and further refine the manufacturing process for large-scale clinical deployment.

## 6. Conclusions

This paper presents the design, modeling, and numerical validation of a compact AMC-backed flexible UHF RFID tag antenna, specifically designed for on-body biomedical and wearable sensing applications. Human tissue proximity typically causes severe detuning, radiation efficiency degradation, and increased specific absorption rate (SAR) for conventional RFID tag antennas.

To address these limitations, an AMC metasurface based on a modified Jerusalem-cross geometry is employed, miniaturized through meandered arms and interdigitated comb-like features. The design achieves an ultra-compact footprint of 0.0246 λ^2^ (32.12 mm × 64.24 mm), representing a 50% reduction in size over traditional AMC unit cells. In addition, the implementation on a custom-tailored, high-permittivity, silicon-doped biocompatible substrate ensures the device is both ultra-thin (2.23 mm) and mechanically conformable for wearable use. Full-wave simulations demonstrate that the AMC structure serves as an effective electromagnetic shielding element, isolating the radiator from lossy human tissue. This isolation yields a gain enhancement of approximately 13 dB (exceeding an order-of-magnitude improvement over conventional standalone on-body tags) and a corresponding extension of the read range. Concurrently, the structure suppresses backward radiation and reduces SAR values by more than tenfold, ensuring strict compliance with user safety standards. The designed tag antenna also exhibits excellent platform tolerance and mechanical robustness, maintaining stable electrical performance across bending radii ranging from 75 mm to 150 mm. By striking a strategic balance between mechanical flexibility, compact dimensions, and high radiation efficiency, this design provides a reliable foundation for non-invasive, high-fidelity epidermal sensing and real-time health monitoring within the evolving IoT-enabled healthcare landscape.

## Figures and Tables

**Table 1:** Comparison with the State-of-the-Art for AMC-backed wearable antennas.

Ref.	f_0_ (MHz)	Substrate (ϵr)	Height (mm)	Area (λ^2^)	Gain On Body (dBi)	Gain on Body with AMC (dBi)	Δ Gain (dB)	Flexible	AMC Sub.	Biocompatible	Air Gap	Platform Tolerant
[42]	2450 2550 2560	Felt (1.3)	2 mm + 7 mm foam	0.0267	0.36 (skin) −2.94 (3-layer) −4.20 (4-layer) −3.45 (arm)	4.13 4.05 4.21 3.15	(stable) 6.6 dBi	Yes	Yes	Yes	7 mm	Yes *
[43]	2450	cotton (1.6)	3.8	0.1925	5.72	7.09	1.37	Yes	Yes	Yes	3 mm	Yes *
[44]	2400	Flexible denim (1.7)	3	0.2304	−1.2	6.98	8.18	Yes	Yes	Yes	1 mm	Yes *
[45]	865 3450 4100	FR4 (4.2)	6.52 + 1.52	0.0362	4.55 5.03 5.6/3.15	4.61 6.24 3.26	1.21	No	No	No	No	No
[46]	5800	Jeans (2)	3.2	2.014	0.99	4.88	5.3	Yes	Yes	Yes	No	Yes
[49]	868	6.24	2.8	0.0283	−15.3	-	-	No	No	Yes	No	No
[50]	915	4.4	6.4	0.422	−2.44	5.01	7.45	No		Yes	No	
[37]	868	2.8	4.18	8.8/103	−18.1	-	-	No	No	Yes	No	No
This Work	868	doped Silicon (6.24)	2.23	0.0246	−21 dB	−8 dB	13 dB	Yes	Yes	Yes	No	Yes

* Claims tolerance but requires specific spacing or air gaps to maintain resonance.

**Table 2:** Electromagnetic parameters and thickness of the multilayer human body model (at 866 MHz).

Material (Bio-Tissue)	Thickness (mm)	Permittivity ϵ_r_	Conductivity σ ([S/m])	Density ρ ([kg/m^3^])
Skin	2	41.3	0.89	1100
Fat	20	5.46	0.05	910
Muscle	37	55.0	0.94	1041
Bone	21	20.8	0.34	1850
Internal Organs	84	52.1	0.91	1000

**Table 3:** SAR Values on Human Model (W/Kg) *.

Tissue Weight	1 g	10 g
Tag Placed directly on Phantom	5.76	1.25
Tag placed on tailored AMC	0.284	0.0864

* Values normalized to 1 W of accepted power to allow standardized comparison with literature.

## Data Availability

Data are available only under request.
